# Case Report: Nerve Root Entrapment Due to Epidural Fibrosis in a Patient With Failed Back Surgery Syndrome: Value of 2-^18^F-Fluorodeoxyglucose Simultaneous Positron Emission Tomography-Magnetic Resonance Imaging

**DOI:** 10.3389/fmed.2022.860545

**Published:** 2022-04-25

**Authors:** Yueh-Hsun Tsai, Guo-Shu Huang, Chi-Tun Tang, Wei-Chou Chang, Yi-Chih Hsu

**Affiliations:** ^1^Department of Surgery, Tri-Service General Hospital, National Defense Medical Center, Taipei City, Taiwan; ^2^Department of Radiology, Tri-Service General Hospital, National Defense Medical Center, Taipei City, Taiwan; ^3^Department of Medical Research, Tri-Service General Hospital, National Defense Medical Center, Taipei City, Taiwan; ^4^Department of Neurosurgery, Tri-Service General Hospital, National Defense Medical Center, Taipei City, Taiwan

**Keywords:** epidural fibrosis, magnetic resonance imaging, failed back surgery syndrome, positron emission tomography, case report

## Abstract

Failed back surgery syndrome (FBSS) is a highly prevalent condition in patients after spine surgery. Although magnetic resonance imaging (MRI) is the gold standard for the diagnosis of epidural fibrosis, it is sometimes difficult to determine if epidural fibrosis contributes to radiculopathy. Herein, we share our experience in locating radiculopathy lesions using simultaneous positron emission tomography (PET)/MRI. 2-[^18^F]-FDG (^18^F-fluorodeoxyglucose) simultaneous PET/MRI maps of body glucose metabolism detected using PET can be used to correlate anatomical details provided by MRI to offer a very clear picture of neural inflammation due to extensive epidural fibrosis. More applications of 2-[^18^F]-FDG simultaneous PET/MRI in low back pain and other musculoskeletal diseases should be further investigated in the future.

## Introduction

Failed back surgery syndrome (FBSS) is defined as persistent back or leg pain after one or more lumbar disk surgeries (1). Treatment for FBSS is often ineffective because of the difficulty in identifying the etiology (2). Magnetic resonance imaging (MRI) is the gold standard for evaluating spine lesions; contrast-enhanced MRI can differentiate between epidural fibrosis, spinal stenosis, and disk herniation (3). Although substantial intra-observer and near-perfect inter-observer agreement have been achieved for the evaluation of epidural fibrosis using MR images (4), we are still unable to determine whether epidural fibrosis causes radiculopathy (5). Positron emission tomography (PET)/computed tomography(CT) has been previously used to detect peripheral nerve pathologies, including neurolymphomatosis and neuritis (6–8). However, to our knowledge, the PET-MR findings of radiculitis after laminectomy have not been reported. Studies using PET to assess metabolic activity showed increased binding in the spinal cord and compressed nerve roots in patients with radicular pain (9). We present a patient with FBSS who presented with refractory back pain radiating to the right leg and for whom root entrapment by epidural fibrosis was observed using PET/MRI.

## Case Report

**FIGURE 1:**
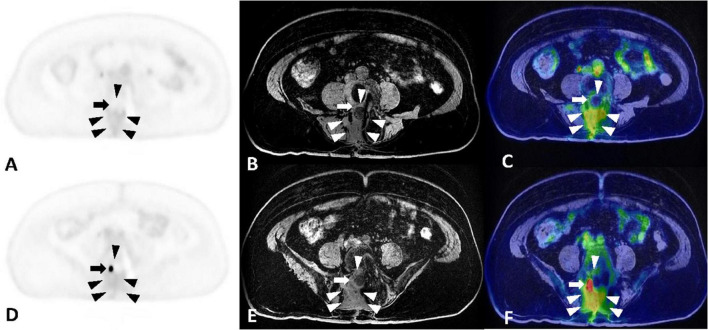
**(A–C)** Maximum intensity projection, MRI and PET-MR fusion images at the level of L3-L4, separately. **(D–F)** Maximum intensity projection, MRI and PET-MR fusion images at the level of L5-S1, separately. Fat-suppression T1-weighted sequences in the axial plane without contrast showing increased and homogeneous intensity within the epidural space [arrowheads in panels **(B,E)**], indicative of extensive scar formation. The fusion images [arrowheads in panels **(C,F)**] reveal mild FDG uptake in the epidural scar with SUVmax of 2.0. The epidural fibrosis encircles the right L4 root [arrow in panel **(C)**] and right S1 root [arrow in panel **(F)**]. The FDG uptake of the right L4 root is similar to that of the adjacent epidural scar, while the right S1 root shows intense FDG uptake with SUVmax of 4.2.

**FIGURE 2:**
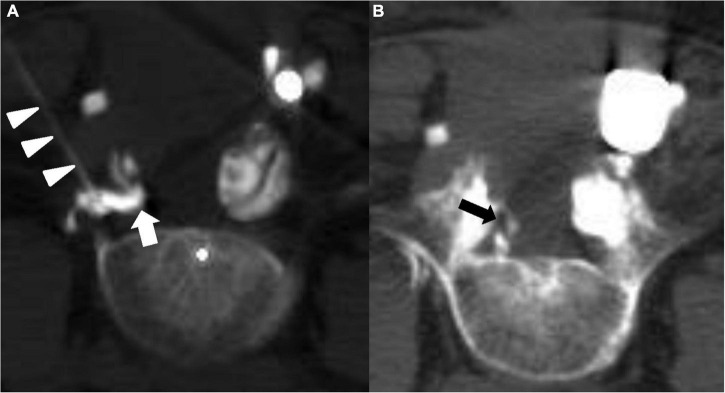
CT-guided transforaminal injection at the level of L5-S1. Axial CT for the extent of contrast spread. **(A)** Visualization of the needle in the epidural space (arrowheads) and the dye (thick arrow) blocked by the scar. **(B)** Contrast filling around the right S1 root (thick arrow) at the ventral epidural space.

The patient was a 66-year-old woman with chronic lower back pain. She denied any history of having systemic diseases, such as diabetes mellitus. She failed from conservative treatment including anti-inflammatories, muscle relaxants, analgesics and physical therapies. After a series of conservative treatments, she underwent, laminectomy of L5-S1 and posterior lumbar interbody fusion of L4–S1 was performed 20 months ago. Persistent back pain with downward radiation at the right foot (predominantly tightness and numbness) was noted after the operation; it correlated to the L5 or S1 dermatome. MRI revealed extensive scars over the sides of L4 to S1, bilaterally. However, the decision for surgical intervention was difficult to make because the patient only had symptoms on the right side. She underwent right pedicle screw removal and decompression but did not have any improvement after surgery. She was also treated with radiofrequency ablation and nerve block, but the effect was only transient and it lasted less than one day. The lower back pain and right leg claudication worsened after 3 months, with a visual analog scale (VAS) score of 9. The patient visited our outpatient department due to persistent symptoms. 2-[^18^F]-FDG PET-MRI scans were arranged and performed on a 3T PET/MRI scanner (Signa PET/MRI, GE Healthcare, Waukesha, WI, United States), which combines 3T MRI with time-of-flight capable silicone photomultiplier-based PET. The result revealed a faint FDG uptake (standard uptake value [SUV] = 1–2) over the right lateral epidural scar at the level of L5/S1; the scar was located along the course of the right S1 root, which revealed a nodular FDG uptake (maximal SUV = 4.2). Multiple epidural scars were seen over the sides of L4 to S1, bilaterally; however, no abnormal FDG uptake other than at the right S1 root was observed. The tentative diagnosis was entrapment neuropathy secondary to epidural fibrosis (Figure 1). We arranged CT-guided transforaminal injection of a lidocaine and contrast dye mixed injectate for the pre-operative diagnosis of right s1 root entrapment by an epidural scar (Figure 2). There was a good response to lidocaine injection for 2–3 hours, however, the symptoms recurred the following day. The diagnosis of epidural fibrosis with right S1 root entrapment was confirmed.

## Discussion

Failed back surgery syndrome has become a common problem in recent years, given the increasing rate of spine operations to treat back pain due to the aging population. It was found in a systematic review that the number of patients reporting recurrent back or leg pain after discectomy ranged from 5 to 36% (10). The post operative pain at the beginning can be caused by the degenerative changes, hematoma formation in the epidural or subdural space, infection, nerve injury. The progression of these etiologies can lead to formation of the epidural fibrosis, which worsened the symptoms due to traction of the fibrotic tissue on adjacent nerve roots. Epidural fibrosis is reported as a contributing factor to postoperative pain in 20–36% of FBSS cases (11). Although patients with extensive epidural fibrosis were shown to be 3.2 times more likely to experience recurrent radicular pain than those with less extensive epidural fibrosis in a previous study (4). we are still unable to determine whether radiculopathy is caused by epidural fibrosis (5).

Positron emission tomography is a sensitive tool for identifying hypermetabolic activity in cells, with broad utility in cancer surveys (12). PET has unique capabilities to provide quantitative information about the molecular and metabolic activity of tissues, but a higher resolution of anatomic information is needed to localize lesions (13). Hence, simultaneous PET/MRI has been more valuable because of the integration of metabolic activity and the detailed anatomy of the spinal root (9). Simultaneous PET/MRI provides better information about soft-tissue, spinal cord, and spinal disk abnormalities than PET/CT (14). The clinical applications of PET/MRI in the musculoskeletal system have been recently reported (13). A study reported the role of PET in identifying inflammation in peripheral nerves in various neuropathies (8) in which multiple epidural scars were seen over the sides of L4 to S1, bilaterally. However, these findings did not correlate with the patient’s clinical symptoms. The decision for surgical intervention was difficult to make because of the difficulty in identifying the primary site of the radiculopathy. PET/MRI in our case revealed increased FDG uptake at the right S1 root, which was correlated to the epidural scar found entrapping the right S1 root on MRI. Epidural injections were administered using a fluoroscopic-guided transforaminal approach at the level of the involved nerve root to confirm the primary lesion, as described in a previous study.

In our case, contrast enhanced MRI was not performed. According to previous study (15), although contrast enhancement in MRI improved the visualization of nerve roots lying in scar tissue among 16% cases and better defined the extent of epidural fibrosis. However, it rarely altered final diagnosis. In our cases, the epidural fibrosis over was clearly defined in L3-L4 and L5-S1 with non-contrast MRI. But only PET/MRI revealed the distinct nerve root lesion. Study performed by Passavanti et al. (16) also concluded that Gadolinium enhanced MRI images compared with unenhanced MRI increased diagnostic confidence and agreement only in differentiating epidural fibrosis from disk herniation mainly in the first 6 months After 18 months, Gadolinium enhanced MRI did neither improve confidence nor agreement. In our case, the patient received the spine surgery 20 months before the PET/MRI scan. Therefore, whether performing a gadolinium MRI should show no difference.

Our case is the first to identify neural inflammation due to extensive epidural fibrosis after laminectomy. Using PET/MRI maps, body glucose metabolism detected using PET to correlate anatomical details provided by MRI can offer a very clear picture of the neural inflammation caused by extensive epidural fibrosis (9). In our experience, simultaneous PET/MRI can aid in the diagnosis of radiculitis in a patient with FBSS, not only for anatomic evaluation, but also for the metabolic assessment of the spinal roots in extensive epidural fibrosis.

## Data Availability Statement

The original contributions presented in the study are included in the article/Supplementary Material, further inquiries can be directed to the corresponding author.

## Ethics Statement

The studies involving human participants were reviewed and approved by the Institutional Review Board of Tri-Service General Hospital. The patients/participants provided their written informed consent to participate in this study. Written informed consent was obtained from the individual(s) for the publication of any potentially identifiable images or data included in this article.

## Author Contributions

Y-HT and W-CC reviewed the literature and contributed to manuscript drafting and revision. G-SH analyzed and interpreted the imaging findings. C-TT and Y-CH were responsible for the revision of the manuscript. All authors issued final approval for the version to be submitted.

## Conflict of Interest

The authors declare that the research was conducted in the absence of any commercial or financial relationships that could be construed as a potential conflict of interest.

## Publisher’s Note

All claims expressed in this article are solely those of the authors and do not necessarily represent those of their affiliated organizations, or those of the publisher, the editors and the reviewers. Any product that may be evaluated in this article, or claim that may be made by its manufacturer, is not guaranteed or endorsed by the publisher.
