# Supplementary material for: Case Report: Nerve Root Entrapment Due to Epidural Fibrosis in a Patient With Failed Back Surgery Syndrome: Value of 2-18F-Fluorodeoxyglucose Simultaneous Positron Emission Tomography-Magnetic Resonance Imaging
Source: Front Med (Lausanne). 2022 Apr 25;9:860545. doi: 10.3389/fmed.2022.860545 (PMC9085244; doi:10.3389/fmed.2022.860545)
Supplement: Supplementary file 1 [file Table_1.DOCX]

**CARE Checklist – 2016: Information for writing a case report**

**Topic Item Checklist item description Line/Page**

**Title 1** The words “case report” should be in the title along with the area of focus 1/1

**Key Words 2** Four to seven key words—include “case report” as one of the key words 14/1

**Abstract 3a** Background: What does this case report add to the medical literature? 16/1

**3b** Case summary: chief complaint, diagnoses, interventions, and outcomes 20/1

**3c** Conclusion: What is the main “take-away” lesson from this case? 24/1,

**Introduction 4** The current standard of care and contributions of this case—with references (1-2 paragraphs) 27/1

**Timeline 5** Information from this case report organized into a timeline (table or figure) 67/2

**Patient Information 6a** De-identified demographic and other patient or client specific information 44/2

**6b** Chief complaint—what prompted this visit? 47/2

**6c** Relevant history including past interventions and outcomes 46/2

**Physical Exam 7** Relevant physical examination findings 53/2

**Diagnostic 8a** Evaluations such as surveys, laboratory testing, imaging, etc. 55/2

**Assessment 8b** Diagnostic reasoning including other diagnoses considered and challenges 57/2

**8c** Consider tables or figures linking assessment, diagnoses and interventions 57/2

**Interventions 9a** Types such as life-style recommendations, treatments, medications, surgery 62/2

**9b** Intervention administration such as dosage, frequency and duration 62/2

**9c** Note changes in intervention with explanation 62/2

**9d** Other concurrent interventions 62/2

**Follow-up and 10a** Clinician assessment (and patient or client assessed outcomes when appropriate) 64/2

**Outcomes 10b** Important follow-up diagnostic evaluations 64/2

**10c** Assessment of intervention adherence and tolerability, including adverse events 64/2

**Discussion 11a** Strengths and limitations in your approach to this case 96/3

**11b** Specify how this case report informs practice or Clinical Practice Guidelines (CPG) 82/3

**11c** How does this case report suggest a testable hypothesis? 97/3

**11d** Conclusions and rationale 100/3

**Patient Perspective 12** When appropriate include the assessment of the patient or client on this episode of care n/a

**Informed Consent 13** Informed consent from the person who is the subject of this case report is required by most journals _______

**Additional Information 14** Acknowledgement section; Competing Interests; IRB approval when required 103/3 262/15
